# Integrated prevention of mother-to-child transmission for human immunodeficiency virus, syphilis and hepatitis B virus in China

**DOI:** 10.2471/BLT.14.139626

**Published:** 2014-11-03

**Authors:** Ai-Ling Wang, Ya-Ping Qiao, Lin-Hong Wang, Li-Wen Fang, Fang Wang, Xi Jin, Jie Qiu, Xiao-Yan Wang, Qian Wang, Jiu-Ling Wu, Sten H Vermund, Li Song

**Affiliations:** a National Center for Women and Children’s Health, Chinese Center for Disease Control and Prevention, Beijing, China.; b National Center for Chronic and Noncommunicable Disease Control and Prevention, Chinese Center for Disease Control and Prevention, Beijing, China.; c National Health and Family Planning Commission of the People’s Republic of China, No.1, Xizhimen South Road, Xicheng District, Beijing, China.; d Vanderbilt Institute for Global Health and Department of Pediatrics, Vanderbilt University School of Medicine, Nashville, United States of America.

## Abstract

**Problem:**

China continues to face challenges in eliminating mother-to-child transmission of human immunodeficiency virus (HIV), syphilis and hepatitis B virus (HBV).

**Approach:**

In 2010, a programme that integrated and standardized prevention of mother-to-child transmission (PMTCT) efforts for HIV, syphilis and HBV was implemented in 1156 counties. At participating antenatal care clinics, pregnant women were offered all three tests concurrently and free of charge. Further interventions such as free treatment, prophylaxis and testing for mothers and their children were provided for HIV and syphilis.

**Local setting:**

China’s national PMTCT HIV programme started in 2003, at which time there were no national programmes for perinatal syphilis and HBV. In 2009, the rate of maternal-to-child transmission of HIV was 8.1% (57/702). Reported congenital syphilis was 60.8 per 100 000 live births. HBV infection was 7.2% of the overall population infected.

**Relevant changes:**

Between 2010 and 2013 the number of pregnant women attending antenatal care clinics with integrated PMTCT services increased from 5.5 million to 13.1 million. In 2013, 12.7 million pregnant women were tested for HIV, 12.6 million for syphilis and 12.7 million for HBV. Mother-to-child transmission of HIV fell to 6.7% in 2013. Data on syphilis transmission are not yet available.

**Lessons learnt:**

Integrated PMTCT services proved to be feasible and effective, and they are now part of the routine maternal and child health services provided to infected women. The services are provided through a collaboration between maternal and child health clinics, the national and local Centers for Disease Control and Prevention, and general hospitals.

## Résumé

**Problème:**

La Chine est toujours confrontée à des difficultés dans l'élimination de la transmission mère-enfant du virus de l'immunodéficience humaine (VIH), de la syphilis et du virus de l'hépatite B (VHB).

**Approche:**

En 2010, un programme qui intégrait et harmonisait les efforts de la prévention de la transmission mère-enfant (PTME) pour le VIH, la syphilis et le VHB, a été mis en œuvre dans 1156 pays. Au moment de bénéficier de soins prénatals, les femmes enceintes se sont vues proposer l'ensemble des trois tests en même temps et gratuitement. Des interventions supplémentaires telles que le traitement gratuit, la prophylaxie et le dépistage pour les mères et leurs enfants, ont été incluses dans le programme.

**Environnement local:**

Le programme national chinois de PTME pour le VIH a débuté en 2003, au moment où il n'y avait encore aucun programme pour le dépistage périnatal de la syphilis et du VHB. En 2009, le taux de transmission mère-enfant pour le VIH était de 8,1% (57/702). Le nombre de cas de syphilis congénital signalés s'élevait à 60,8 pour 100 000 naissances vivantes. Le taux d'infection par le VHB était de 7,2% de l'ensemble de la population infectée.

**Changements significatifs:**

Entre 2010 et 2013, le nombre de femmes enceintes bénéficiant de soins prénatals avec des services de PTME intégrés a augmenté de 5,5 millions à 13,1 millions. En 2013, 12,7 millions de femmes enceintes ont été testées pour le VIH, 12,6 millions pour la syphilis et 12,7 millions pour le VHB. La transmission mère-enfant pour le VIH a chuté à 6,7% en 2013.

**Leçons tirées:**

Les services de PTME intégrée ont montré leur faisabilité et leur efficacité. Ils font maintenant partie des pratiques courantes des services de soins maternels et pédiatriques proposées aux femmes infectées. Ces services sont proposés en collaboration avec les cliniques de santé, les centres nationaux et locaux pour le contrôle et la prévention des maladies et les hôpitaux généraux.

## Resumen

**Situación:**

China sigue haciendo frente a desafíos en la eliminación de la transmisión maternoinfantil del virus de la inmunodeficiencia humana (VIH), la sífilis y el virus de la hepatitis B (VHB).

**Enfoque:**

En 2010 se puso en marcha en 1156 condados un programa que integraba y estandarizaba los esfuerzos de prevención de la transmisión maternoinfantil (PTMI) para el VIH, la sífilis y el VHB. En las clínicas de atención prenatal que participaron se ofrecieron a las mujeres embarazadas las tres pruebas al mismo tiempo y de forma gratuita. El programa incluyó otras intervenciones como el tratamiento gratuito, profilaxis y pruebas para las madres y sus hijos.

**Marco regional:**

El programa nacional de PTMI del VIH en China comenzó en 2003, momento en el que no había ningún programa nacional para la sífilis y el VHB perinatales. En 2009, la tasa de transmisión maternoinfantil del VIH fue del 8,1 % (57/702). La sífilis congénita notificada fue de 60,8 por cada 100 000 nacidos vivos, y la infección por el VHB fue del 7,2 % de la población general infectada.

**Cambios importantes:**

Entre 2010 y 2013, el número de mujeres embarazadas que asistieron a clínicas de atención prenatal con servicios integrados de PTMI aumentó de 5 450 000 a 13 070 000. En 2013, 12 720 000 mujeres embarazadas se sometieron a la prueba del VIH, 12 600 000 a la prueba de la sífilis y 12 730 000 a la del VHB. La transmisión maternoinfantil del VIH se redujo al 6,7 % en 2013.

**Lecciones aprendidas:**

La PTMI integrada demostró ser factible y eficaz, y ha pasado a formar parte de los servicios rutinarios de salud maternoinfantil que se proporcionan a las mujeres infectadas. Se ofrece a través de una colaboración entre las clínicas, los Centros para el Control y la Prevención de Enfermedades locales y nacionales y los hospitales generales.

## ملخص

**المشكلة:**

لا تزال الصين تواجه تحديات في التخلص من سريان فيروس العوز المناعي البشري (HIV) والزهري وفيروس التهاب الكبد "باء" (HBV) من الأم إلى الطفل.

**الأسلوب:**

في عام 2010، تم تنفيذ برنامج لإدراج وتوحيد جهود الوقاية من سريان فيروس العوز المناعي البشري والزهري وفيروس التهاب الكبد "باء" من الأم إلى الطفل (PMTCT) في 1156 مقاطعة. وعٌرض على النساء الحوامل في عيادات الرعاية السابقة للولادة المشاركة، إجراء كل الاختبارات الثلاثة بشكل متزامن وبالمجان. وتم إدراج تدخلات أخرى مثل العلاج المجاني والتوقية وفحص الأمهات وأطفالهن في البرنامج.

**المواقع المحلية:**

بدأ البرنامج الوطني للوقاية من سريان فيروس العوز المناعي البشري من الأم إلى الطفل في الصين عام 2003، ولم يكن هناك حينها برامج وطنية للزهري وفيروس التهاب الكبد "باء" في الفترة المحيطة بالولادة. وفي عام 2009، كان معدل سريان فيروس العوز المناعي البشري من الأم إلى الطفل 7.8 % (أعداد مطلقة). وكان الزهري الخلقي الذي تم الإبلاغ عنه 60.8 لكل 100 ألف وليد حي. وكانت العدوى بفيروس التهاب الكبد "باء" 7.2 % من السكان المصابين بشكل عام.

**التغيّرات ذات الصلة:**

في الفترة من عام 2010 إلى عام 2013، ارتفع عدد النساء الحوامل اللاتي حضرن إلى عيادات الرعاية السابقة للولادة التي أدرجت خدمات الوقاية من السريان من الأم إلى الطفل من 5.45 مليون إلى 13.07 مليون امرأة. وفي عام 2013، تم فحص 12.72 مليون امرأة حامل للتحقق من فيروس العوز المناعي البشري و12.60 مليون امرأة حامل للتحقق من الزهري و12.73 مليون امرأة حامل للتحقق من فيروس التهاب الكبد "باء". وانخفض سريان فيروس العوز المناعي البشري من الأم إلى الطفل إلى 6.7 % في عام 2013.

**الدروس المستفادة:**

ثبت أن إدراج الوقاية من السريان من الأم إلى الطفل مجدٍ وفعال، و هي الآن جزء من الخدمات الروتينية لصحة الأمومة والطفولة ويتم تقديمها للأمهات المصابات. ويتم تقديم الخدمات بالتعاون مع العيادات الصحية والمراكز الوطنية والمحلية للوقاية من الأمراض ومكافحتها والمستشفيات العامة.

## 摘要

**问题:**

中国继续面临消除艾滋病病毒（HIV）、梅毒和乙型肝炎病毒（HBV）母婴传播的挑战。

**方法:**

2010年一项综合标准化HIV、梅毒和HBV母婴传播预防工作的工作在1156个县实施。在参与该工作的产前保健诊所中，为孕妇同时免费提供所有三项检测。工作包含进一步的干预措施，诸如母亲及其孩子免费治疗、预防和检测。

**当地状况:**

中国全国预防艾滋病毒母婴传播工作（PMTCT HIV）始于2003年，当时还没有围产期梅毒和乙型肝炎病毒的国家项目。2009年，母婴HIV传播的比例是8.1% (57/702)。据报告，每10万例活产中先天性梅毒有60.8例。HBV感染占总人口7.2%。

**相关变化:**

在2010年和2013年间，在提供整合艾滋病毒母婴传播预防服务的产前保健诊所获得保健服务的孕妇从5.5万增加到13.1万人。在2013年，12.7万名孕妇接受了HIV检测，12.6万名孕妇接受梅毒检测，12.7万名孕妇接受HBV检测。2013年，HIV母婴传播降低至6.7%。

**经验教训:**

实践证明，整合预防艾滋病毒母婴传播可行有效，目前是常规妇幼保健服务的组成部分。服务由健康诊所、全国和地方疾病预防控制中心以及综合性医院合作提供。

## Резюме

**Проблема:**

Перед Китаем по-прежнему стоит проблема устранения передачи вируса иммунодефицита человека (ВИЧ), сифилиса и вируса гепатита В (ВГВ) от матери к ребенку.

**Подход:**

В 2010 году в 1156 уездах страны была реализована программа, которая интегрировала и стандартизировала усилия по профилактике передачи от матери к ребенку (ППМР) таких заболеваний как ВИЧ, сифилис и ВГВ. В антенатальных клиниках, участвующих в программе, беременным женщинам одновременно и бесплатно предлагалось пройти все три теста. В эту программу также были включены дополнительные мероприятия, такие как бесплатное лечение, профилактика и тестирование матерей и их детей.

**Местные условия:**

Национальная программа ППМР для ВИЧ-инфекции в Китае была запущена в 2003 году, когда еще не существовало национальных программ по профилактике перинатальной передачи сифилиса и ВГВ. В 2009 году уровень передачи ВИЧ от матери к ребенку составлял 8,1% (57/702). Врожденный сифилис был зарегистрирован у 60,8 из 100 000 живорожденных детей. Уровень ВГВ составлял 7,2% от общей численности инфицированного населения.

**Осуществленные перемены:**

В период между 2010 и 2013 гг. число беременных женщин, посещавших антенатальные клиники, предлагающие комплексные услуги по ППМР, увеличилось с 5,5 млн. до 13,1 млн. В 2013 году 12,7 млн. беременных женщин прошли тестирование на ВИЧ, 12,6 млн. — на сифилис и 12,7 млн. — на ВГВ. В 2013 году уровень передачи ВИЧ-инфекции от матери ребенку снизился до 6,7%.

**Выводы:**

Комплексные услуги по ППМР оказались реализуемыми и эффективными и в настоящее время входят в плановые услуги по обеспечению здоровья матери и ребенка, оказываемые инфицированным женщинам. Эти услуги предоставляются в сотрудничестве с поликлиниками, Центрами по контролю и профилактике заболеваний на местном и национальном уровнях и больницами общего профиля.

## Introduction

The World Health Organization (WHO) has called for integrated action to eliminate new paediatric human immunodeficiency virus (HIV) infection and congenital syphilis by 2015.^1^^,^^2^ However, in 2009, mother-to-child transmission of these diseases was still high in China.

At the end of 2009, the HIV prevalence in China was 0.057% (range: 0.042–0.071), with an estimated 740 000 people (range: 560 000–920 000) living with HIV.^3^ Before the antiretroviral therapy era, the mother-to-child transmission rate was estimated to be 34.8%.^4^ In 2009, this rate fell to 8.1% after widespread HIV screening of pregnant women, and HIV infected mothers were offered free antiretroviral prophylaxis for HIV and replacement feeding to avoid breast feeding their children.

Syphilis, caused by a spirochete bacterium, was seldom reported in the Chinese population two decades ago, however the incidence has increased to 24.7 cases per 100 000 people in 2009.^5^ Reported congenital syphilis has increased from 25.5 per 100 000 live births in 2005 to 60.8 per 100 000 live births in 2009.^5^

In addition, hepatitis B virus (HBV) is a public health problem. In 2006, 7.2% of the Chinese population aged 1–59 years were hepatitis B surface antigen positive.^6^ In 2010, China’s Ministry of Health began to integrate and standardize prevention of mother-to-child transmission (PMTCT) efforts for HIV, syphilis and HBV. Subsequently, the ministry initiated comprehensive services nationwide. In this report, we describe China’s implementation of these integrated PMTCT services.

## Local setting

Supported by the Government of China, the national PMTCT programme for HIV was initiated in 2003 and covered 453 counties, cities and districts by 2009. At the participating antenatal care clinics, free HIV screening was offered for pregnant women. The interventions for HIV-infected mothers included HIV-tailored midwifery services, free antiretroviral therapy or prophylaxis and practical guidance on infant feeding. Exposed infants received free antiretroviral medicines, HIV antibody testing at 12 and 18 months of age and developmental monitoring. In 2003 there were no nationwide programmes designed to prevent maternal transmission of syphilis and HBV.

## Approach

To meet the WHO goals for elimination of paediatric HIV infection and congenital syphilis by 2015, China faced many national challenges. In the second half of 2010, the Government of China incorporated a nationwide PMTCT programme for HIV, syphilis and HBV into the existing maternal and child health-care system.

In February 2011, the Ministry of Health issued the *Protocol for prevention of mother-to-child transmission of HIV, syphilis and hepatitis B*, which contained the government’s response strategy, intervention measures, and requirements regarding organization and management.^7^ The protocol provided guidance to all regions for implementation of integrated PMTCT efforts for HIV, syphilis and hepatitis B. It also reiterated the official policy of strengthening government leadership, ensuring that all departments fulfil their respective responsibilities, promoting full social mobilization and broad participation, integrating service resources, improving intervention quality, expanding coverage and promoting standardization.^7^ From 2010 to 2013, the central government allocated over 3.4 billion Yuan (573 million United States dollars), to implement these services.

For PMTCT, testing and counselling are the first intervention steps within the antenatal care environment. In 2010, health-care facilities providing midwifery and antenatal care services that participated in the programme at provincial, prefecture, county and township levels started to offer free HIV, syphilis and hepatitis B counselling and testing for pregnant women. Free PMTCT interventions were provided to all infected mothers and their children throughout the nation.

The interventions for HIV were identical to interventions in the original national PMTCT programme for HIV, except that the current programme offered early HIV diagnosis for exposed infants at 6–8 weeks of age.

Mothers infected with syphilis were provided free treatment with 2.5 million units of intramuscular benzathine penicillin once a week for three weeks and appropriate midwifery services to prevent transmission during labour. Exposed infants were provided with free prophylaxis with benzathine penicillin and offered testing for syphilis every three months up to 18 months of age. For infected infants, free treatment was offered.

Children born to women testing positive for hepatitis B surface antigen were provided with free hepatitis B immunoglobulin (100 IU) within 24 hours after birth and three hepatitis B vaccinations within 24 hours of birth, at 1 month and 6 months of age, in accordance with national guidelines.^8^ Testing and treatment of children were not covered by the programme.

The programme supported training of health-care providers. Between 2010 and 2012 training-of-trainers was provided yearly at national and provincial level on the elements of the PMTCT care cascade. The training lasted for approximately three days. Subsequently, at provincial and prefectural level, the instructors held yearly training sessions, that normally lasted for 1–3 days, for local health-care providers to improve programme capacity. Health-care providers were trained on implementation of integrated PMTCT interventions and management of infected pregnant women and their children. They were also trained in data collection. Staff were trained in data management and analysis. The National Center for Women and Children’s Health incorporated all PMTCT data on HIV, syphilis and HBV into the pre-existing management information system of the national PMTCT programme.

## Relevant changes

By 2013 the programme covered 41% (1156/2853) of counties, cities and districts within 31 provinces, autonomous regions and municipalities in China. In six provinces, autonomous regions and municipalities with the most serious epidemics, all counties were covered.

The PMTCT programme reached 13.1 million pregnant women in 2013, 8.7 million more than in 2009. Of these, 12.7 million were tested for HIV antibodies, representing an increase in testing rates from 85.4% in 2009 to 97.3% in 2013. There was a 63.1% increase in pregnant women diagnosed with HIV, from 3662 cases in 2009 to 5973 cases in 2013. Mother-to-child transmission of HIV fell from 8.1% (57/702) in 2009 to 6.7% (145/2180) in 2013. In the same year, 12.6 million pregnant women in the programme were also tested for syphilis, covering 96.4% of women reached by the integrated programme. We commenced data collection on mother-to-child transmission of syphilis in the latter half of 2012. To be able to exclude transmission, syphilis-infected mothers and their children are followed until the children are 18 months of age. Therefore, we do not yet have transmission data for syphilis. Hepatitis B surface antigen testing was successfully done in 97.4% (12.7 million) pregnant women. Among neonates born to women testing positive for hepatitis B surface antigen, 97.7% received hepatitis B immunoglobulin (Table 1).

**Table 1:** The programme for integrated prevention of mother-to-child transmission of human immunodeficiency virus, syphilis and hepatitis B virus, China, 2009–2013

Characteristic	Year				
	2009	2010	2011	2012	2013
No. of counties	453	1 156	1 156	1 156	1 156
Millions of pregnant women attending antenatal care	4.38	5.45	9.39	12.07	13.07
Millions of pregnant women tested for HIV (%)	3.74 (85.39)	4.84 (88.81)	8.73 (92.97)	11.64 (96.44)	12.72 (97.32)
No. of pregnant women diagnosed with HIV (%)	3 662 (0.10)	4 146 (0.09)	5 313 (0.06)	5 779 (0.05)	5 973 (0.05)
No. of children acquiring HIV through transmission from an HIV-infected mother (%)^a^	57 (8.12)	86 (7.90)	124 (7.41)	131 (7.05)	145 (6.65)
Millions of pregnant women tested for syphilis (%)	NA	NA	7.30^b,c^ (84.98)	11.48 (95.11)	12.60 (96.40)
No. of pregnant women diagnosed with syphilis (%)^b^	NA	NA	14 822 (0.20)	24 307 (0.21)	30 520 (0.24)
Millions of pregnant women tested for Hep B virus surface antigen (%)	NA	NA	7.67^c^ (89.29)	11.72 (97.10)	12.73 (97.40)
No. of Hep B virus exposed neonates that received Hep B immunoglobulin (%)	NA	NA	301 048 (86.21)	599 071 (94.42)	774 916 (97.74)

Hep B: Hepatitis B; HIV: human immunodeficiency virus; NA: not available.

^a^ For 2009 *n* = 702; 2010 *n* = 1088; 2011 *n* = 1673; 2012 *n* = 1858; 2013 *n* = 2180.

^b^ Definition of syphilis seropositivity: both nontreponemal antigen serologic test and *Treponema pallidum* antigen serologic test are positive.

^c^ In 2011, in the first year of implementation, HIV, syphilis and HBV testing were not integrated in all programme sites, hence the coverage of screening for syphilis and HBV was less than that of HIV. The number of pregnant women covered by syphilis and HBV testing was 8.59 million.

## Lessons learnt

Integrated prevention of three vertically-transmitted diseases – HIV, syphilis and HBV – proved to be feasible and effective at a large scale in China. Pregnant women were offered all three tests concurrently, free of charge. The programme was fully nested within the existing maternal and child health-care system in China at county, township, and village levels. Pregnant women infected with HIV, syphilis or HBV were immediately enrolled for integrated PMTCT programme services as a part of their routine antenatal, postnatal, and children’s care. Maternal and child health clinics, the national and local Centers for Disease Control and Prevention, and general hospitals collaborated well, which led to a successful implementation of the programme (Box 1). The integrated PMTCT programme has helped China meet the WHO goal.

Box 1 **Summary of main lessons learnt**

- Integrated prevention of mother-to-child transmission proved to be feasible and effective for HIV, syphilis and hepatitis B virus in China.
- This programme was implemented through the current maternal and child health-care system in China, improving its sustainability.
- Collaboration between maternal and child health clinics/hospitals, the national and local Centers for Disease Control and Prevention, and general hospitals were crucial for the programme’s success.

Implementing the integrated programme involved challenges including shortage of human resources, quality of services, and suboptimal availability of services in some areas. To address the concerns about availability, the Government of China has expanded the integrated PMTCT programme to 1638 counties in August 2014, using the same strategy that had been deployed in 2010. This expansion will allow more pregnant women to access free integrated PMTCT services. Improving the capacity of health-care staff and quality-associated monitoring and evaluation through further training will be our future focus.

In conclusion, the expansion of the integrated PMTCT programme both in content and coverage can significantly contribute towards achieving the dual goal of eliminating paediatric HIV infection and congenital syphilis, as well as addressing the ongoing burden of perinatal HBV transmission. Our experience took place in a large population with high disease burdens. The Chinese model may be of interest to other nations that seek to better integrate HIV, syphilis and HBV services into the broader maternal and child health context.
